# Impact of Antiphase Boundaries on Structural, Magnetic and Vibrational Properties of Fe_3_Al

**DOI:** 10.3390/ma13214884

**Published:** 2020-10-30

**Authors:** Martin Friák, Miroslav Černý, Monika Všianská, Mojmír Šob

**Affiliations:** 1 Institute of Physics of Materials, v.v.i., Czech Academy of Sciences, Žižkova 22, CZ-616 62 Brno, Czech Republic; cerny.m@fme.vutbr.cz (M.Č.); 230038@mail.muni.cz (M.V.); mojmir@ipm.cz (M.Š.); 2 Central European Institute of Technology (CEITEC), Brno University of Technology, Technická 2, CZ-616 69 Brno, Czech Republic; 3 Faculty of Mechanical Engineering, Brno University of Technology, Technická 2, CZ-616 69 Brno, Czech Republic; 4 Department of Chemistry, Faculty of Science, Masaryk University, Kotlářská 2, CZ-611 37 Brno, Czech Republic; 5 Central European Institute of Technology, CEITEC MU, Masaryk University, Kamenice 5, CZ-625 00 Brno, Czech Republic

**Keywords:** Fe-Al, antiphase boundaries, magnetism, ab initio, stability, phonons

## Abstract

We performed a quantum-mechanical study of the effect of antiphase boundaries (APBs) on structural, magnetic and vibrational properties of Fe${}_{3}$Al compound. The studied APBs have the {001} crystallographic orientation of their sharp interfaces and they are characterized by a 1/2〈111〉 shift of atomic planes. There are two types of APB interfaces formed by either two adjacent planes of Fe atoms or by two adjacent planes containing both Fe and Al atoms. The averaged APB interface energy is found to be 80 mJ/m${}^{2}$ and we estimate the APB interface energy of each of the two types of interfaces to be within the range of 40–120 mJ/m${}^{2}$. The studied APBs affect local magnetic moments of Fe atoms near the defects, increasing magnetic moments of Fe${}^{\mathrm{II}}$ atoms by as much as 11.8% and reducing those of Fe${}^{I}$ atoms by up to 4%. When comparing phonons in the Fe${}_{3}$Al with and without APBs within the harmonic approximation, we find a very strong influence of APBs. In particular, we have found a significant reduction of gap in frequencies that separates phonon modes below 7.9 THz and above 9.2 THz in the defect-free Fe${}_{3}$Al. All the APBs-induced changes result in a higher free energy, lower entropy and partly also a lower harmonic phonon energy in Fe${}_{3}$Al with APBs when compared with those in the defect-free bulk Fe${}_{3}$Al.

## 1. Introduction

Antiphase boundaries (APBs) are rather common extended defects appearing in crystals with ordered sublattices. They are formed when two parts of the crystal are shifted one with respect to the other. The APBs can be created when two grains crystallizing from the melt have each of them a different origin of the lattice and the difference is not a multiple of translational vectors of the superlattices (so-called thermal APBs as they form at higher temperatures). Dislocations can also create APBs (at any temperature) during their motion through an ordered phase when their Burgers vectors are not translation vectors of the ordered superlattice (so-called deformation APBs).

Our computational study focuses on APBs in Fe${}_{3}$Al as an intermetallic compound belonging into a very promising class of Fe-Al-based materials exhibiting several interesting properties, such as relatively low density, remarkable resistance to oxidation or low cost of raw materials [1,2,3,4,5,6,7,8]. Due to these properties, they have been in recent decades intensively studied both experimentally (see, e.g., Refs. [9,10,11,12,13,14,15,16,17,18,19]) and theoretically (see, e.g., Refs. [20,21,22,23,24,25,26,27,28,29,30,31,32,33,34,35,36,37,38,39,40]).

**Figure 1:**
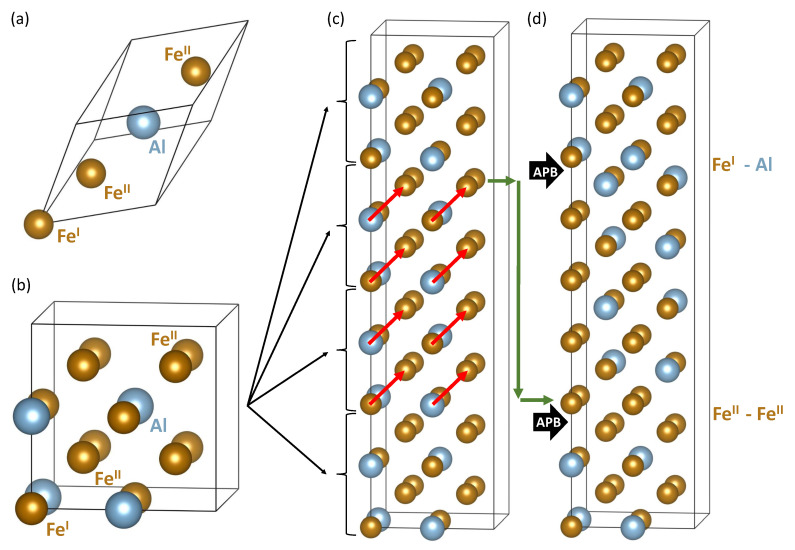
Visualizations of (super-)cells that were used in our theoretical study. Part (**a**) shows a rhombohedral primitive unit cell of Fe${}_{3}$Al with 4 atoms and includes a naming convention of Fe sublattices when Fe${}^{\mathrm{II}}$ sites are twice more abundant than the Fe${}^{I}$ sites. Part (**b**) exhibits a 16-atom cube-shaped elementary cell containing 4 formula units. A 64-atom supercell, as an 1 × 1 × 4 multiple of the 16-atom elementary cell, is visualized in part (**c**) and includes red vectors defining a shift characterizing the studied type of antiphase boundaries (APBs). When applying the APB shift to the central half of the 64-atom supercell in part (c) an APB-containing supercell shown in part (**d**) is formed. To (i) keep the stoichiometry and (ii) apply the 〈111〉 shift to all atoms in the middle half of (c), one atomic plane is cyclically shifted as schematically marked by a series of green arrows (an APB shift applies also to this plane). There are two APB interfaces per each 64-atom supercell shown in part (c). One is formed by two adjacent layers containing both Fe${}^{I}$ and Al atoms (the Fe${}^{I}$-Al APB interface) and the second one consisting of two planes of Fe${}^{\mathrm{II}}$ atoms (the Fe${}^{\mathrm{II}}$-Fe${}^{\mathrm{II}}$ APB interface).

Regarding APBs in iron aluminides, two types of APBs were experimentally found in the D0${}_{3}$ superlattice using transmission electron microscopy (TEM) techniques [41,42]. The first one is defined by a shift of the interfacing grains in the 〈100〉 direction by a half of the lattice parameter defined for a 16-atom cube-shaped D0${}_{3}$ cell (see Figure 1b) and is associated solely with the D0${}_{3}$ superlattice (D0${}_{3}$-type of APBs). The other type of APBs that we focus on in the present study can appear both in the D0${}_{3}$ superlattice and in the B2 lattice (it is called a B2-type of APBs). The B2-type is formed when two parts of the crystal are shifted in the 〈111〉 direction by a half of the lattice parameter of the B2 cell containing 2 atoms. In the case of deformation APBs, both the above mentioned types separate partials of superdislocations in Fe-Al alloys [43,44,45] as the dislocations alter mutual position of sublattices and atomic planes when moving through the crystal. Other APB-related findings are in Refs. [46,47,48,49,50].

We studied the D0${}_{3}$-APBs, which are specific to the D0${}_{3}$ superlattice, in (i) Fe${}_{3}$Al with and without Cr additions [51] and (ii) Fe-Al-Ti compounds [52]. In our current research we build upon our previous expertise with the B2-type of APBs that we theoretically examined earlier in Fe${}_{70}$Al${}_{30}$ alloy [53].

## 2. Methods

All our ab initio calculations were performed using the Vienna Ab initio Simulation Package (VASP) [54,55] that implements the density functional theory [56,57]. We employed projector augmented wave (PAW) pseudopotentials [58,59] and the generalized gradient approximation (GGA) in the parametrization according to Perdew and Wang [60] (PW91) in combination with the Vosko-Wilk-Nusair correction [61] for the exchange and correlation energy. This set-up correctly predicts the ground state of Fe${}_{3}$Al when the D0${}_{3}$ structure is preferred over the L1${}_{2}$ structure by about 5.5 meV/atom [62]. We used 64-atom, 192-atom and 256-atom supercells built as multiples of the 16-atom cube-shaped cell of Fe${}_{3}$Al (shown in Figure 1b). The 64-atom supercells are 1 × 1 × 4 multiples (see examples in Figure 1c,d) of the 16-atom cell and 192-atom supercells are 1 × 1 × 12 multiples of the 16-atom cell. Finally, the biggest 256-atom supercells were generated by the PHONOPY software [63] as 2 × 2 × 1 multiples of the 64-atom supercells (2 × 2 × 4 multiples of the 16-atom cell of Fe${}_{3}$Al) and were used for phonon calculations. The plane-wave energy cut-off was equal to 400 eV and the product of the number of Monkhorst-Pack k-points and the number of atoms was equal to 27,648 (e.g., 12 × 12 × 3 k-point mesh in the case of 64-atom supercells in Figure 1c,d). All studied supercells were fully relaxed (with respect to their atomic positions, cell shape as well as the volume) and the forces were reduced under 0.001 eV/Å in the case of 64-atom supercells and under 0.006 eV/Å in the case of 192-atom 1 × 1 × 12 supercells. In particular, the residual forces were smaller than 0.001 eV/Å for those atomic configurations for which phonon spectra were computed. All local magnetic moments were initially set up as ferromagnetic.

## 3. Results

We start with our results related to a defect-free Fe${}_{3}$Al bulk. We determined its equilibrium lattice parameter (of 16-atom cube-shaped unit cell shown in Figure 1b) to be equal to 5.731 Å. This value is very close to both theoretical value 5.738 Å listed in Ref. [32] (when using the Full-Potential Linearized-Augmented-Plane-Wave (FLAPW) method and Perdew-Burke-Ernzerhof (PBE) approximation [64] to the exchange-correlation energy) and experimental data, 5.792 Å, also mentioned in Ref. [32]. Local magnetic moments of Fe atoms are different due to their different environments. The Fe${}^{I}$ atoms (that have 8 Fe${}^{\mathrm{II}}$ atoms in their first coordination shell, 1NN) and Fe${}^{\mathrm{II}}$ atoms (with 4 Al and 4 Fe${}^{I}$ in their 1NN) are found to be equal to 2.374 $\mu_{B}$ and 1.868 $\mu_{B}$, respectively. These values are very close to theoretical FLAPW-PBE values (2.39 and 1.90 $\mu_{B}$) [32] and qualitatively agree with experimental data (2.18/2.16 and 1.50/1.46 $\mu_{B}$) Ref. [32].

**Figure 2:**
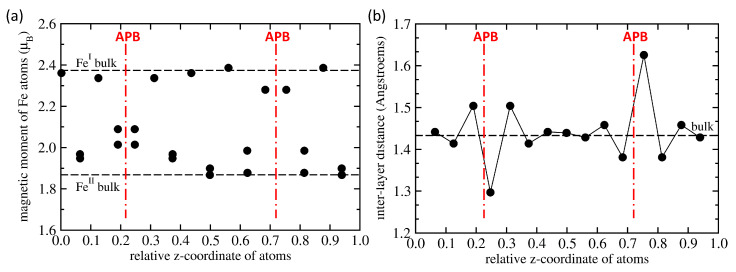
Local magnetic moments of Fe atoms (**a**) and inter-layer distances (**b**) for the 64-atom supercell with the examined antiphase boundaries. The values are compared with those in the bulk (horizontal dashed lines) and the positions of APBs are schematically indicated by red vertical dashed-dotted lines. The line in part (b) connecting the data points is added only to guide the eye.

Next we focus on the APB-containing Fe${}_{3}$Al. It is worth noting that the APB-containing 64-atom supercell (shown in Figure 1d) preserves the stoichiometry of Fe${}_{3}$Al but there are two different APB interfaces in the supercell. One type of APB interface located in the lower half of Figure 1d is formed by two adjacent planes of Fe${}^{\mathrm{II}}$ atoms while the other type, which is shown in the upper half of Figure 1d, is formed by two adjacent planes containing Fe${}^{I}$ and Al atoms. Consequently, some quantities that we compute, such as the APB interface energy, represent an average over the two types of APB interfaces, for others we have local information for each interface or even atom. Regarding atom-resolved quantities, we summarize local magnetic moments of Fe atoms and inter-layer distances in Figure 2a,b, respectively. Each type of iron atoms, i.e., either Fe${}^{I}$ or Fe${}^{\mathrm{II}}$, responds to the presence of the APB differently but, as it was mentioned above, each of the two types of APB interfaces is formed by different type of Fe atoms. Regarding the local magnetic moments in Figure 2a, all data points above 2.2 $\mu_{B}$ are those related to the Fe${}^{I}$ atoms, while those below 2.2 $\mu_{B}$ correspond to the twice-more abundant Fe${}^{\mathrm{II}}$ atoms. Local atomic relaxations partly lift the degeneracy of Fe${}^{\mathrm{II}}$ atoms and pairs of values are seen for closely similar values of *z*-coordinate in Figure 2a. When comparing the calculated values with those in the bulk (see the black horizontal dashed lines in Figure 2a) it is obvious that the APBs (i) increase the local magnetic moments of Fe${}^{\mathrm{II}}$ atoms (in particular at the Fe${}^{\mathrm{II}}$-Fe${}^{\mathrm{II}}$ APB interface) by up to 11.8 % (up to 2.089 $\mu_{B}$ from 1.868 $\mu_{B}$) and (ii) decrease the magnetic moments of Fe${}^{I}$ atoms (in particular at the Fe${}^{I}$-Al APB interface) by about 4 % (down to 2.28 $\mu_{B}$ from 2.374 $\mu_{B}$).

As seen in Figure 2a, the impact of APBs includes one or two layers of iron atoms apart from APB interfaces. The local magnetic moments of Fe atoms in the middle of the 64-atom supercell, which are furthest from the APB interfaces (e.g., $\mu^{I}$ = 2.386 $\mu_{B}$ for *z* = 0.5602 and $\mu^{\mathrm{II}}$ = 1.899 $\mu_{B}$ for *z* = 0.4984), are very similar to those in the bulk ($\mu^{I}$ = 2.374 $\mu_{B}$ and $\mu^{\mathrm{II}}$ = 1.868 $\mu_{B}$). This we interpret so that our 64-atom supercell is big enough to eliminate most of spurious interactions between periodic images.

As far as the inter-planar distances are concerned, Figure 2b shows for each atomic plane the difference between its *z*-coordinate and that of the adjacent atomic plane below (with a lower *z*-coordinate). The computed values are again compared with those for the APB-free bulk (see the black horizontal dashed line in Figure 2b). The Fe${}^{\mathrm{II}}$-Fe${}^{\mathrm{II}}$ APB interface causes a very significant contraction of the inter-layer distance between the interfacing Fe-only atomic planes while a weaker and sort-of damped-oscillating effect is obtained for the planes further away from this APB type. In contrast, the Fe${}^{I}$-Al APB interface causes a very significant expansion of the inter-layer distance between the interfacing Fe${}^{I}$-Al atomic planes while a weaker and damped-oscillating effect is obtained for the planes further away from this APB type. Again, it is worth noting that inter-layer distance in the middle of the supercell in between both APB interfaces (equal to 1.43927 Å for *z* = 0.4982) is indeed very close to that in the bulk (1.43316 Å) as evidence that our 64-atom supercell has a sufficient size.

**Figure 3:**
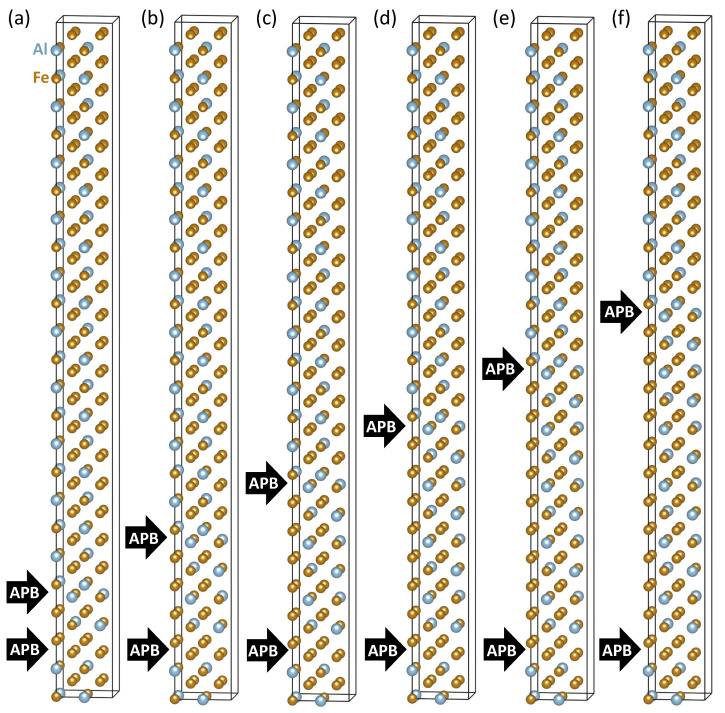
Schematic visualizations of 192-atom supercells (1 × 1 × 12 multiples of the 16-atom cell shown in Figure 1b) that we used for examining possible long-range interactions between the two APB interfaces. The studied Fe${}^{\mathrm{II}}$-Fe${}^{\mathrm{II}}$ and Fe${}^{I}$-Al APB interfaces were separated by one 16-atom Fe${}_{3}$Al unit, i.e., 4 atomic planes (**a**), two 16-atom Fe${}_{3}$Al units (**b**), three 16-atom Fe${}_{3}$Al units (**c**), four 16-atom Fe${}_{3}$Al units (**d**), five 16-atom Fe${}_{3}$Al units (**e**) and six 16-atom Fe${}_{3}$Al units, i.e., 24 atomic planes (**f**).

To further examine the strength of interactions between periodic images of the studied APB interfaces, we used 3 times bigger 192-atom 1 × 1 × 12 supercells and located the two APB interfaces in a series of different distances, see Figure 3. We count the separation between the center of Fe${}^{\mathrm{II}}$-Fe${}^{\mathrm{II}}$ APB interface and the center of the Fe${}^{I}$-Al APB interface similarly as before in multiples of 16-atom Fe${}_{3}$Al unit (4 atomic planes) but this16-atom cell is no longer cube-shaped but weakly tetragonal (see below). The separation in the *z*-direction was set from a minimum of one multiple of 16-atom Fe${}_{3}$Al unit in Figure 3a to maximum of six multiples of 16-atom Fe${}_{3}$Al unit (24 atomic planes) in Figure 3f. The distance to the nearest periodic image was six or more multiples of 16-atom Fe${}_{3}$Al unit.

**Figure 4:**
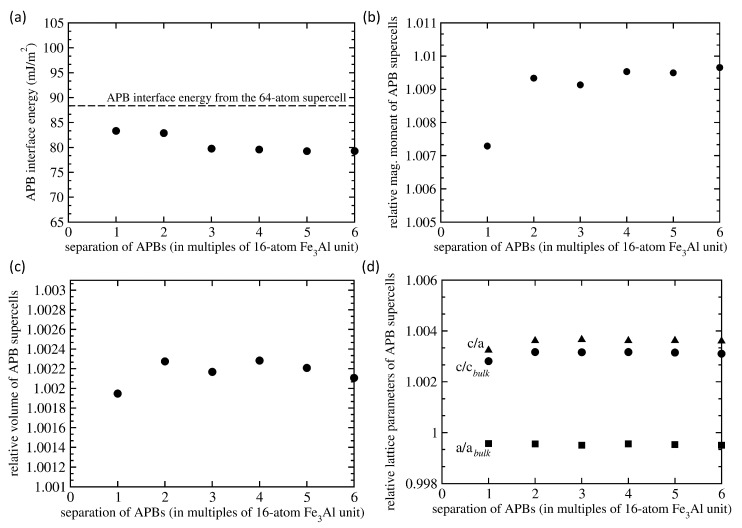
Computed properties of 192-atom 1 × 1 × 12 supercells of Fe${}_{3}$Al with different separation between the two APB interfaces (see Figure 3). In particular we present the APB interface energy (averaged over both types of interfaces) in part (**a**), the total magnetic moment relative to the magnetic moment of the same amount of bulk Fe${}_{3}$Al without any APBs (**b**), a relative volume with respect to that of APB-free Fe${}_{3}$Al bulk (**c**) and lattice parameters (and their ratio) again relative to the corresponding values in the APB-free Fe${}_{3}$Al bulk (**d**). The averaged APB interface energies obtained using the 192-atom supercells are compared with the value computed using the 64-atom supercell (shown in Figure 2d), which has the same separation of APBs interfaces but three times lower separation of periodically repeated images (black horizontal dashed line).

Our calculations for the 192-atom 1 × 1 × 12 supercells indicate that the averaged APB interface energy 〈γ〉 is about 10 % lower than the value of 88 mJ/m${}^{2}$ obtained from the calculations in which we used 64-atom supercell shown in Figure 1d. The calculated averaged APB interface energies are shown in Figure 4a. With increasing separation of the two types of interfaces a converged value is about 80 mJ/m${}^{2}$. The 10% reduction in 〈γ〉 found in bigger supercells may seem as rather big change but the actual values are, in fact, rather small themselves. Considering that the value of 88 mJ/m${}^{2}$ represents 5.7 meV per atom energy difference between the 64-atom supercells, the 10% change is close to an expected error bar of our calculations.

It is worth noting that calculations of APB interface energies for each type of APB interface composition would require either non-stoichiometric supercells or stoichiometric (but vacuum-containing) slabs. We prefer to avoid these calculations as they introduce another type of defects (either the off-stoichiometry or surfaces). Fortunately, using a few additional thermodynamic considerations we can at least estimate APB interface energies of both types of studied APBs. In particular, we assume that both interface energies γ(Fe${}^{I}$-Al) and γ(Fe${}^{\mathrm{II}}$-Fe${}^{\mathrm{II}}$) are positive. An extreme range of these interface energies is then from 0 to 160 mJ/m${}^{2}$ when one type of extreme value, i.e., 0 or 160 mJ/m${}^{2}$, for one type of the interface would require the other type of the interface with the other extreme value, i.e., 160 or 0 mJ/m${}^{2}$, respectively, in order to have the averaged value equal to 80 mJ/m${}^{2}$. Well, we do not expect that the APB interface energy can drop to 0 mJ/m${}^{2}$ because the APBs would then form extremely easily and that is not observed in experiments. Therefore, we speculate that a narrower but still realistic range of interface energies is from 40 to 120 mJ/m${}^{2}$ for both γ(Fe${}^{I}$-Al) and γ(Fe${}^{\mathrm{II}}$-Fe${}^{\mathrm{II}}$) APB interfaces.

Regarding the magnetic properties, relative differences of total magnetic moment per 192-atom supercells with respect to the same amount of APB-free bulk Fe${}_{3}$Al is shown in Figure 4b. The total magnetic moment is found to increase only very slightly by less than 1% for the most distant APB interfaces. The computed volume (again relative to the defect-free bulk) is visualized in Figure 4c and the individual lattice parameters *a* and *c* in Figure 4d. Only very small changes are found to be induced by well separated APB interfaces. The structural defect-related changes are important for the stability of defects because significant changes in the volume would induce significant strains and stresses close to the defect or in the defect-free matrix surrounding the defect. These strains would then cause additional elastic strain energies that would be added to the computed interface energies. However, the APBs increase the volume by only about 0.2%. This small change is likely due to the fact that the APB-containing lattice slightly shirks in directions within the interface plane, i.e., lattice parameter *a*, but slightly expands in the direction perpendicular to the APB interfaces (the *c* lattice parameter).

**Figure 5:**
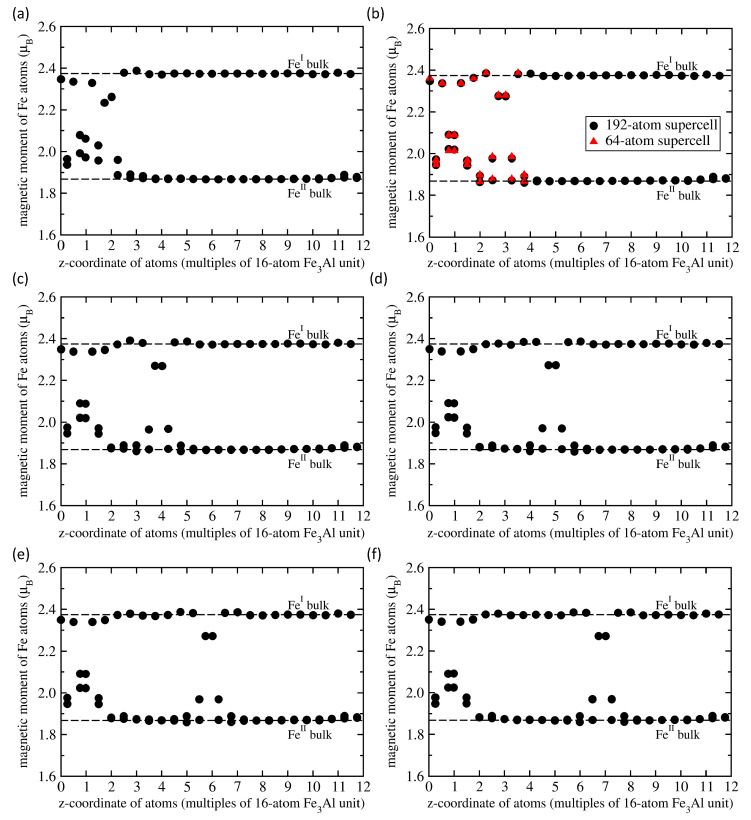
Calculated local magnetic moments of Fe atoms in 192-atom 1 × 1 × 12 supercells of Fe${}_{3}$Al with the APB interfaces (shown in Figure 3) separated by one 16-atom Fe${}_{3}$Al unit (**a**), two units (**b**), three (**c**), four (**d**), five (**e**) and six 16-atom Fe${}_{3}$Al units (**f**). For the separation of 2 units in part (b), the computed values (black circles) are compared with those (red triangles) shown in Figure 2a obtained for the 64-atom supercell presented in Figure 1d that has the same distance between the APBs interfaces.

There is one more aspect related to the magnetism that should be discussed. While the APB-induced changes in the total magnetic moment are very small, less than 1%, see Figure 4b, the changes of local magnetic moments of individual atoms are much bigger, as shown in Figure 5. The calculated results in Figure 5 show that the magnetic moments of Fe${}^{I}$ atoms are either reduced or very slightly increased while those of Fe${}^{\mathrm{II}}$ atoms are either increased or very slightly reduced.

The small change in the value of the total magnetic moment is caused by nearly perfect compensation of these positive and negative changes. The series of sub-figures in Figure 5 for the pair of APB interfaces separated by different distances indicates that each of the APB interfaces induces rather specific pattern of changes in the neighboring Fe atoms. While this pattern is not well recognizable in the case of APB interfaces separated by only four atomic planes in Figure 5a, it is nearly identical for other distances between the interfaces in Figure 5b–f. In particular, the local magnetic moments of Fe atoms in a 192-atom supercell summarized in Figure 3b as black circles in Figure 5b are accompanied by the values (red triangles) that were obtained for the Fe atoms in the 64-atom supercell shown in Figure 1d. These values are also presented in Figure 2a. The overlap of these two data sets neatly illustrates the fact that already the 64-atom 1 × 1 × 4 supercell is big enough for computing the properties of the studied APBs (it also means that the impact of APBs on local magnetic moments is rather localized).

**Figure 6:**
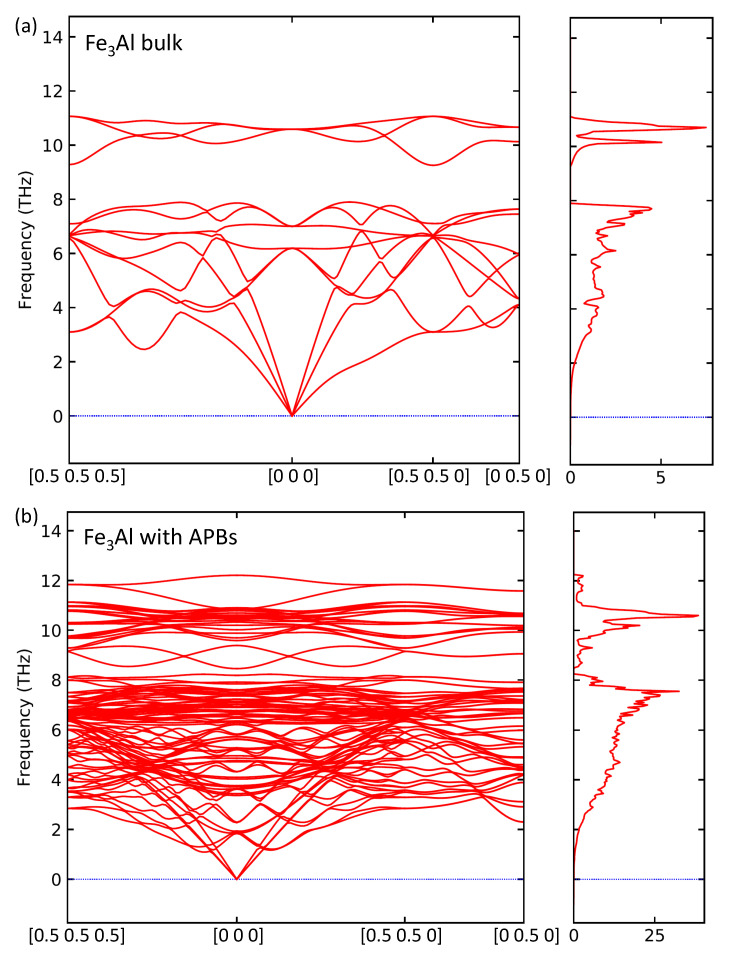
Calculated phonon frequencies and the corresponding densities of vibrational states for the defect-free Fe${}_{3}$Al (**a**) and Fe${}_{3}$Al with the studied two types of APB interfaces (**b**).

Having the above-discussed confirmation that the 64-atom 1 × 1 × 4 supercell is big enough for our computational purposes, we also analysed APB-related changes induced in vibrational properties. The PHONOPY software [63] generated in total 64 atomic displacements for the 64-atom supercell shown in Figure 1d. We computed forces due to these atomic displacements using 256-atom 2 × 2 × 1 multiples of the 64-atom supercell, i.e., 2 × 2 × 4 multiples of the 16-atom cell. The calculations were performed employing (i) an additional support grid for the evaluation of the augmentation charges (in VASP) and (ii) symmetrization of forces (in PHONOPY). The resulting phonon spectra and densities of phonon states for both defect-free Fe${}_{3}$Al (the supercell shown in Figure 1c) and the 64-atom supercell with APBs shown in Figure 1d are presented in Figure 6a,b, respectively.

As far as the phonons of defect-free Fe${}_{3}$Al are concerned, the main features excellently agree with the experimental data for 300 K reported in Refs. [65,66]. First, the most importantly, the defect-free Fe${}_{3}$Al is found mechanically stable with respect to any phonon mode studied.

Second, the frequencies of modes, which cover the range between about 9.2 THz and 11 THz, are separated by a relatively large gap from phonon modes under 7.9 THz. Our phonon frequencies also agree with previous theoretical calculations, such as presented in Ref. [67]. As far as the corresponding density of vibrational states (phonon DOS or pDOS) is concerned, the main features are consistent with those presented in Ref. [68].

Regarding the phonons for the Fe${}_{3}$Al containing APBs in Figure 6b, there are a few major differences induced by the studied APBs. First and the foremost, the gap in phonon frequencies separating the vibrational and optical modes is nearly closed. Second, the peak at the density of phonon states, which forms the bottom of the frequency gap, is very sharply cut at 7.9 THz in the defect-free Fe${}_{3}$Al while the APBs make the cut at 7.9 THz less sharp by inducing an additional small peak located at (and above) 8 THz. Lastly, the reduction of the overall symmetry leads to a much higher number of phonon branches. The system with APBs is, despite all these changes, found mechanically stable with respect to all studied phonon modes (there are no imaginary frequencies). Both the above mentioned modifications related to the gap in frequencies of phonon modes agree with general trends reported in Ref. [68] where phonons for the ordered and disordered states of ${}^{57}$Fe${}_{3}$Al are compared. These experimental data do not clearly show a gap in phonon frequencies. The reason is, most likely, the fact that also other defects existing in real samples may have a similar impact, i.e., narrowing/closing the gap. The comparison of ordered and disordered states shows, nevertheless, qualitatively the same features. First, an increase of the phonon density of states in the energy range where the theory predicts the gap in phonon frequencies (about 33–42 meV in Figure 14 in Ref. [68] when 1 THz = 4.136 meV). Second, a broadening of the peak at about 8 THz (about 33 meV) was observed. Considering that the studied APB interfaces effectively lower the ordering of the Fe${}_{3}$Al, we predict essentially the very same phenomena.

Having the phonon frequencies ω(qν) determined for each reciprocal-space vector q and mode ν within the harmonic approximation for the static-lattice minimum-energy volume, we next evaluate a few other properties related to vibrational degrees of freedom. In particular, Helmholtz free energy *F*, the entropy *S*, harmonic phonon energy *E* and constant-volume heat capacity $C_{v}$:

$$\begin{array}{ccc} F & = & \mathrm{const}.+\frac{1}{2}\sum_{q\nu }\hbar \omega \left(q\nu \right)+k_{B}T\;\sum_{q\nu }\ln\left[1-\exp\left(-\hbar \omega \left(q\nu \right)/k_{B}T\right)\right],\;\;\;S=-\left(\frac{\partial F}{\partial T}\right),\\ E & = & \sum_{q\nu }\hbar \omega \left(q\nu \right)\left[\frac{1}{2}+\frac{1}{\exp(\hbar \omega \left(q\nu \right)/k_{B}T)-1}\right],\;\;\;C_{v}=-{\left(\frac{\partial E}{\partial T}\right)}_{v}. \end{array}$$

**Figure 7:**
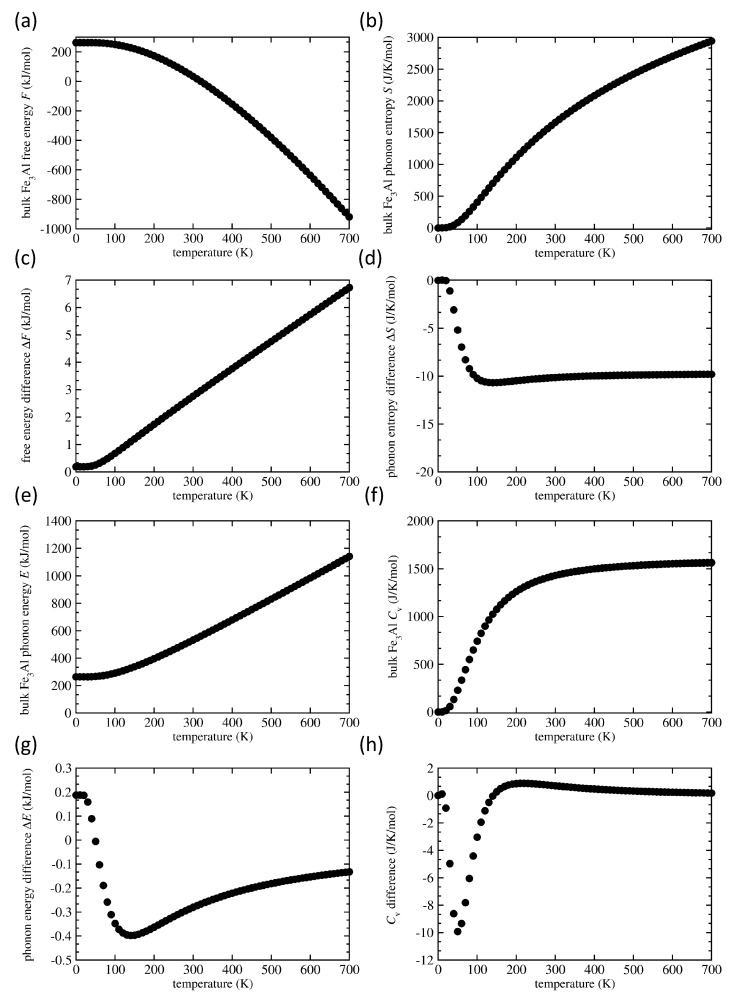
The Helmholtz free energy *F* (**a**) and vibrational entropy *S* (**b**) of the defect-free bulk Fe${}_{3}$Al together with changes induced in them by the studied APBs in (**c**,**d**), respectively. Also shown are the harmonic phonon energy *E* (**e**) and constant-volume heat capacity $C_{v}$ (**f**) of the perfect bulk Fe${}_{3}$Al accompanied by the APB-induced changes in parts (**g**,**h**), respectively. The elementary entity for defining one mol is the 64-atom supercell.

The results for the bulk Fe${}_{3}$Al (without any APBs) and the differences induced by APBs (such as $\Delta F=F\left({\mathrm{Fe}}_{3}\mathrm{Al}-\mathrm{APBs}\right)-F\left({\mathrm{Fe}}_{3}\mathrm{Al}-\mathrm{bulk}\right)$) are summarized in Figure 7 as a function of temperature. The Helmholtz free energy *F* in the defect-free bulk Fe${}_{3}$Al is depicted in Figure 7a. It is higher when APBs are introduced into the system (see Figure 7c) and the difference is growing with increasing temperature as a clear impact of vibrational entropy terms. The free-energy change is a few kJ/mol, i.e., it is rather small because 1 kJ/mol represents in our case about 0.1625 meV/atom energy difference between the two 64-atom supercells (note that the total-energy difference without vibrations induced by both APBs is about 5.7 meV/atom as discussed above). In terms of the averaged APB interface energy, 1 kJ/mol corresponds to a change of about 2.5 mJ/m${}^{2}$. The fact that the change ΔF is positive, i.e., thermal vibrations destabilize the APBs with increasing temperature, is not intuitive but both positive and negative changes were reported earlier for APBs in L1${}_{2}$-structure Ni${}_{3}$Al [69]. The vibrational entropy *S* for the perfect bulk Fe${}_{3}$Al is shown in Figure 7b. The APB-induced changes are negative, i.e., the vibrational entropy of Fe${}_{3}$Al with APBs is lower (see Figure 7d). The harmonic phonon energy *E* for the defect-free bulk in Figure 7e increases with temperature but APB-induced changes are rather complex (see Figure 7g), from positive values below T = 50 K to negative ones above this threshold temperature (at least within the analyzed temperature range up to 700 K).

Lastly, when taking the constant-volume heat capacity of the bulk Fe${}_{3}$Al in Figure 7f as a reference, the APB-related changes (see Figure 7h) are also quite complicated: they turn negative at very low temperature close to T = 0 K and they decrease up to T = 50 K when they reach a minimum, start increasing so that they become positive for temperatures above T = 140 K, reach a maximum at about T = 200 K and then slowly decrease for yet higher temperatures.

## 4. Conclusions

We performed an ab initio study of impact of antiphase boundaries (APBs), which are characterized by a 1/2〈111〉 shift of atomic planes, on structural, magnetic and vibrational properties of Fe${}_{3}$Al compound. The studied APBs have the {001} crystallographic orientation of their sharp interfaces. There are two types of APB interfaces formed by either two adjacent planes of Fe${}^{\mathrm{II}}$ atoms or by two adjacent planes containing both Fe${}^{I}$ and Al atoms. By comparing supercells with the same number of atoms with and without APBs we obtained an averaged APB interface energy equal to 80 mJ/m${}^{2}$. We further estimate that the APB interface energy of each of the two types of interfaces is within the range of 40–120 mJ/m${}^{2}$. The studied APBs significantly affect local magnetic moments of Fe atoms near the defects—they are increased in the case of Fe${}^{\mathrm{II}}$ atoms by as much as 11.8% and reduced in the case of Fe${}^{I}$ atoms by up to 4%. As these changes mutually nearly perfectly compensate, the total magnetic moment increases due to the presence of the studied APBs by less than 1% (in case of 192-atom supercells with well separated APB interfaces of both types). When comparing phonons in the Fe${}_{3}$Al with and without APBs within the harmonic approximation, we find a clear influence of APBs, such as a significant reduction of gap in frequencies that separates phonon modes below 7.9 THz and above 9.2 THz in the defect-free Fe${}_{3}$Al. An overall impact of APBs-induced changes results in a small increase of the free energy, lower vibrational entropy and a lower harmonic phonon energy (above T = 50 K).
